# Effect of Hydride Types on the Fracture Behavior of a Novel Zirconium Alloy Under Different Hydrogen-Charging Current Densities

**DOI:** 10.3390/ma18020467

**Published:** 2025-01-20

**Authors:** Kun Zhang, Hang Fan, Baifeng Luan, Ping Chen, Bin Jia, Pengwan Chen, Hao Wang

**Affiliations:** 1 College of Materials Science and Engineering, Chongqing University, Chongqing 400030, China; zk_330@163.com (K.Z.); bfluan@cqu.edu.cn (B.L.); 2 National Key Laboratory of Nuclear Reactor Technology, Nuclear Power Institute of China, Chengdu 610005, China; hangfan_npic@126.com (H.F.); chenping_npic@163.com (P.C.); 3 State Key Laboratory of Explosion Science and Protection Technology, Beijing Institute of Technology, Beijing 100081, China; bin.jia@bit.edu.cn (B.J.); pwchen@bit.edu.cn (P.C.)

**Keywords:** zirconium alloys, hydrogen embrittlement, hydrides, hydrogen-charging current density

## Abstract

Hydrogen embrittlement is a critical issue for zirconium alloys, which receives long-term attention in their applications. The formation of brittle hydrides facilitates crack initiation and propagation, thereby significantly reducing the material’s ductility. This study investigates the tensile properties and hydride morphology of a novel zirconium alloy under different hydrogen-charging current densities ranging from 0 to 300 mA/cm^2^, aiming to clarify the influence of hydrides on the fracture behavior of the alloy. The mechanical property results reveal that, as the hydrogen-charging current density increases from 0 to 100 mA/cm^2^, the maximal elongation decreases from 24.99% to 21.87%. When the current density is further increased from 100 mA/cm^2^ to 200 mA/cm^2^, the maximal elongation remains basically unchanged. However, a substantial drop in elongation is observed as the hydrogen-charging current density rises from 200 mA/cm^2^ to 300 mA/cm^2^, decreasing from 20.77% to 15.18%, which indicates a marked deterioration in hydrogen embrittlement resistance. Subsequently, phase compositions, fracture morphology, and hydride types in the fracture region of tensile specimens were characterized. The morphology and quantity of hydrides change with increasing hydrogen-charging current density. When the hydrogen-charging current density reaches 100 mA/cm^2^, the δ-phase hydrides form, which significantly reduces the ductility of the zirconium alloy. At a hydrogen-charging current density of 200 mA/cm^2^, metastable ζ-phase hydrides are formed, resulting in negligible variations in the alloy’s mechanical properties. However, when it comes to 300 mA/cm^2^, stable δ-phase hydrides with diverse morphologies form, leading to a pronounced degradation in tensile performance. Finally, by integrating mechanical tests with microstructural characterization, the influence of hydrides formed under different hydrogen-charging current densities on the zirconium alloy was analyzed. With increasing hydrogen-charging current density, the maximal elongation of the specimens gradually decreases, while the tensile strength steadily increases. At a hydrogen-charging current density of 300 mA/cm^2^, a larger amount of hydrides is formed, and the hydride type transitions completely from a mixture of δ-phase and ζ-phase hydrides to entirely δ-phase hydrides. The formation of lath-like δ-phase hydrides induces twinning structures, resulting in further lattice mismatch, which significantly reduces the maximal elongation of the zirconium alloy. Additionally, the morphology of the δ-phase hydrides changes from slender needle-like structures to lath-like structures, leading to a notable increase in internal stress, which in turn further enhances the tensile strength of the specimens.

## 1. Introduction

Zirconium alloys are widely used as fuel cladding materials in nuclear reactors due to their exceptional corrosion resistance, favorable mechanical properties, and low thermal neutron absorption. However, under water-cooled reactor conditions, zirconium alloy cladding readily absorbs hydrogen, leading to the formation of hydrides. This hydride formation induces hydrogen embrittlement, significantly reducing the ductility of the material and posing a critical risk to the safe operation of nuclear fuel systems. As a result, hydrogen embrittlement becomes one of the key challenges limiting the long-term application of zirconium alloys in the nuclear industry [1,2,3,4,5,6]. Understanding the mechanism of hydride formation and its influence on mechanical properties of zirconium alloys is therefore imperative for addressing this issue and improving the safety and reliability of these materials.

The formation of hydrides in zirconium alloys is primarily governed by hydrogen concentration. At ambient temperature, when the hydrogen content is lower than the solubility limit of the α-Zr matrix, hydrogen atoms dissolve interstitially into the tetrahedral sites of the matrix, forming a solid solution with a hexagonal close-packed (HCP) structure. However, when the hydrogen content exceeds the solubility limit, hydrides begin to nucleate and precipitate. The commonly observed hydride phases include ζ-Zr_X_H, γ-ZrH, δ-ZrH_X_, and ε-ZrH_X_ [7,8,9,10,11,12,13]. Kim et al. [14] demonstrated that the maximum stress of zirconium alloy ring tensile tests increases with rising hydrogen content. Specifically, the tensile strength of Zry-4 increased by nearly 50 MPa at a hydrogen content of 700 ppm compared to 0 ppm, though the elongation exhibited a significant decline. Tung et al. [15] investigated the effects of hydrogen content on the mechanical properties of a Zry-4 alloy. Their findings revealed that, at room temperature, specimens with higher hydrogen contents underwent a ductile-to-brittle transition; as the hydrogen content increased from 0 to 712 ppm, the elongation decreased by more than 30%. However, this ductile-to-brittle transition disappeared when the temperature exceeded 100 °C. In contrast, Nagase et al. [16] reported that the strength of zirconium alloy specimens remained nearly constant within the temperature range of 300–573 K. Nevertheless, when the hydrogen content was below 500 ppm, the ductility of the specimens exhibited minimal reduction at these temperatures. In contrast, a significant decline in ductility was observed at 300 K, when the hydrogen content exceeded 600 ppm. Therefore, hydrides formed during the increase in hydrogen content are considered the primary cause of the ductile-to-brittle transition in zirconium alloys. The influence of hydride content, type, morphology, distribution, and orientation is closely related to the material’s mechanical properties [14,17,18,19,20,21,22,23,24,25,26,27,28,29].

Extensive research has been conducted on the characteristics of individual hydride phases. ζ-ZrX_H_, for instance, is a metastable ductile hydride with a structure similar to α-Zr, and often serves as an intermediate phase during the transformation from α-Zr to δ-ZrH_X_, as well as during the dissolution and reprecipitation of δ-ZrH_X_ [26,30]. γ-ZrH, characterized by a face-centered tetragonal (FCT) structure, typically exhibits a needle-like morphology. Its formation mechanism is primarily influenced by annealing temperature and crystallographic orientation [31,32,33]. δ-ZrH_X_, the most commonly observed hydride phase in zirconium alloys, possesses a face-centered cubic (FCC) structure and can be distributed both intragranularly and intergranularly. Due to its high dislocation density, δ-ZrH_X_ exhibits high strength but low ductility, and is classified as brittle hydride [11,18]. Additionally, ε-ZrH_X_ is a low-temperature stable phase with an FCT structure. Its formation is accompanied by a volumetric expansion of up to 20%, which further exacerbates the brittleness of zirconium alloys [27,32]. These studies have provided critical insight into the individual characteristics of hydrides in zirconium alloys.

In practical applications, however, multiple hydride phases often coexist within zirconium alloys. In a Zr-H system, interactions between different hydride phases may involve shear transformations, diffusionless martensitic transformations, and complex phase transitions accompanied by hydrogen diffusion [30,31,32]. Such microstructural phenomena can significantly alter the mechanical response of zirconium alloys and complicate their fracture behavior. For example, ζ-Zr_X_H is considered a metastable phase that plays a pivotal role during the precipitation and dissolution of δ-ZrH_X_ [14,26]. Furthermore, under tensile loading conditions, δ-ZrH_X_ has been observed to transform into γ-ZrH, a brittle hydride, which accelerates the fracture of zirconium alloys [18,24,33]. Despite these findings, relatively limited research has been conducted on the synergistic interactions among multiple hydride phases and their combined effects on the mechanical properties of zirconium alloys. Addressing this research gap is essential for developing a comprehensive understanding of hydrogen embrittlement and for improving the hydrogen resistance of zirconium alloys.

To address this challenge, the present study focuses on a novel zirconium alloy to systematically investigate the effects of hydride types and phase transformation characteristics on fracture behavior under varying hydrogen-charging conditions. Tensile testing was conducted to evaluate the mechanical property variations in the zirconium alloy under different hydrogen-charging conditions. Advanced characterization techniques, including scanning electron microscopy (SEM) and transmission electron microscopy (TEM), were utilized to analyze the microstructure, phase composition, and hydride types of fractured specimens. By correlating fracture behavior with hydride characteristics, this study elucidates the influence of hydride types on the fracture mechanisms of zirconium alloys.

## 2. Experimental Methods

### 2.1. In Situ Hydrogen-Charging Slow Strain Rate Tensile Test

The zirconium alloy is provided by the Nuclear Power Institute, and consists of 0.10–0.40 wt% Fe, 0.80–1.20 wt% Sn, 0.90–1.10 wt% Nb and the balance Zr. It was halved along its diameter and flattened through cold rolling. The flattened samples were heated to 400 °C in a muffle furnace at a heating rate of 10 °C/min and held at this temperature for 240 min, followed by furnace cooling at a cooling rate of 5 °C/min. Dog bone-shaped tensile specimens were prepared with a total length of 47 mm, a gauge section width of 4 mm, and a gauge length of 15 mm for tensile and slow strain rate tensile (SSRT) experiments under dynamic hydrogen charging. Prior to testing, the specimens were polished sequentially using 1000#, 2000#, and 5000# sandpaper to achieve a smooth and flat surface.

**Figure 1:**
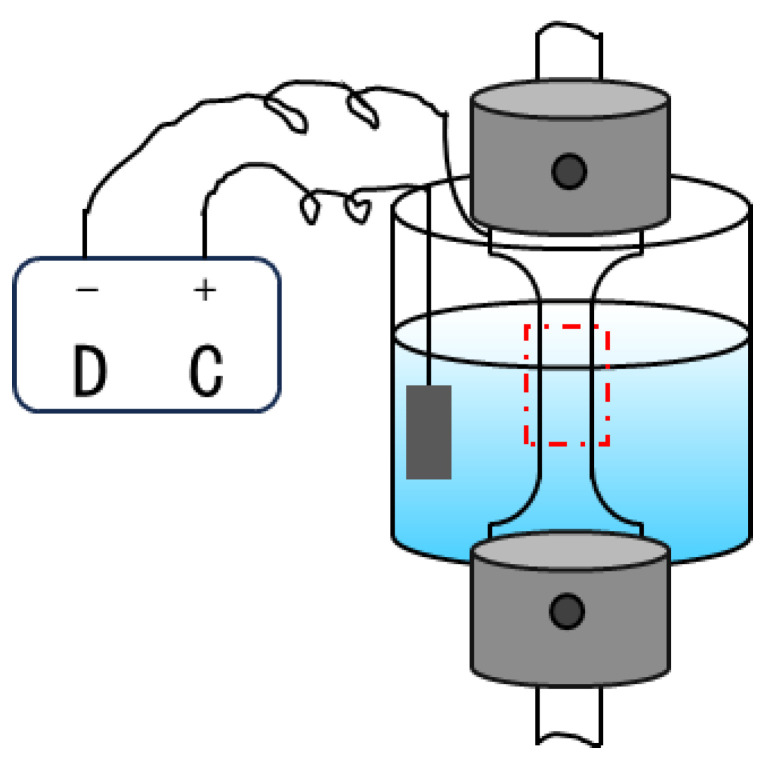
Schematic diagram of in situ hydrogen-charging slow strain rate tensile (SSRT) test (the red box indicates the solution immersion gauge section).

The hydrogen-charging solution used in the dynamic hydrogen charging SSRT experiments consisted of 5% H_2_SO_4_ and 2 g/L thiourea. The hydrogen-charging current densities were set at 0, 100, 200, and 300 mA/cm^2^, with the generated hydrogen typically having a purity higher than 99.9 vol.%. Prior to performing the dynamic hydrogen charging SSRT experiments, the zirconium alloy specimens were acid-cleaned in a solution of 45% H_2_O, 45% HNO_3_, and 10% HF for 30 s to remove the surface oxide layer. Dynamic hydrogen charging SSRT experiments were conducted at a strain rate of 10^−5^ s^−1^, corresponding to a tensile rate of 9 × 10^−3^ mm/min, as illustrated in Figure 1. The values represent the averages obtained from three independent tests.

The DC machine supplies the hydrogenation current, driving the electrochemical hydrogen-charging process. By controlling the current density and stability of the electrochemical reaction, the DC machine ensures that the sample surface continuously and uniformly absorbs hydrogen atoms.

### 2.2. Microstructural Characterization

The gauge section of the tensile-tested samples was extracted and subjected to consistent surface preparation procedures to minimize the potential influences of sample preparation on hydride formation and crack initiation. The surface was first ground using 5000# sandpaper, followed by mechanical polishing with a SiO_2_ suspension. To remove the surface oxide layer and stress-induced layers, electropolishing was subsequently conducted in a solution composed of 10% perchloric acid and 90% ethanol. The electropolishing process was performed at a voltage of 30 V for 30 s. The temperature for electrolytic polishing was set to −30 °C.

The fracture morphology and crack characteristics were examined using an S-4800 scanning electron microscope (SEM) (Hitachi, Tokyo, Japan;). The fractured samples were immersed in a fracture cleaning solution and ultrasonically agitated using an ultrasonic cleaner with a power of 30 W/L and a vibration frequency of 30 kHz for 1.5–2 min to thoroughly clean the corroded fracture surfaces.

The microstructure of the samples was characterized using an FEI Titan Cube 80–300 (FEI, Oregon, United States) transmission electron microscope and selected regions near the fracture site of the tensile-fractured samples for TEM observation. The tensile-tested samples were ground with 2000# sandpaper to a thickness of 80–100 µm, followed by ion thinning. The entire ion thinning process was carried out under a liquid nitrogen environment until completion to ensure that the zirconium alloy did not deform during low-temperature thinning. The tensile-tested samples were used a Gatan 691 ion thinning instrument (Gatan, Shanghai, China) for specimen thinning and electrochemical twin-jet thinning was conducted with a Struers Tenupol-5 apparatus (Struers, Copenhagen, Denmark).

Phase composition analysis was conducted using an X-ray diffractometer (XRD) with a step size of 0.02°, a scanning speed of 2°/min, and a scanning angle range (2θ) of 20–100°.

## 3. Results and Discussion

### 3.1. Stress–Strain Relations

**Figure 2:**
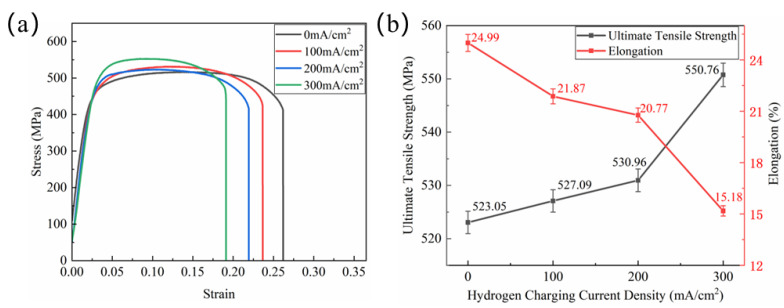
Tensile test results: (**a**) engineering stress–strain curves, (**b**) variation in tensile strength and elongation with hydrogen-charging current density.

Figure 2 displays the tensile test results performed under varying hydrogen-charging current densities. As shown in Figure 2a, when the hydrogen-charging current density is 200 mA/cm^2^ or lower, both the tensile strength and yield strength of the material remain largely unaffected, indicating that the hydride formation has a negligible influence on the mechanical properties. In contrast, at a hydrogen-charging current density of 300 mA/cm^2^, the stress–strain curves exhibit significant deviations, highlighting the pronounced effect of hydrogen on the material’s mechanical behavior.

Figure 2b presents the mechanical properties corresponding to the stress–strain curves shown in Figure 2a, with the data representing the average values obtained from two parallel tests. The tensile strength is defined as the maximum stress observed in the curve, while the maximal elongation is determined as the ratio of the actual displacement to the gauge length. The results show that when the hydrogen-charging current density reaches 300 mA/cm^2^, the tensile strength of the sample increases from 530.96 MPa to 550.76 MPa, whereas the maximal elongation decreases from 20.77% to 15.18%. Prior to this point, both properties exhibit minimal variation. These findings suggest that the effect of hydrogen-charging current density on the mechanical properties of the material is nonlinear.

### 3.2. Fracture Morphology of Post Tensile Test Specimens

**Figure 3:**
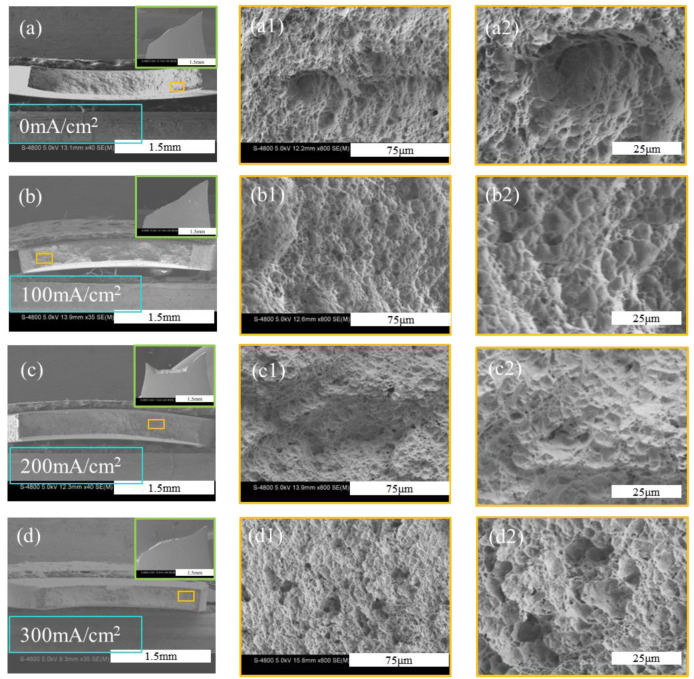
Fracture morphologies under different hydrogen-charging current densities: (**a**) 0 mA/cm^2^, (**b**) 100 mA/cm^2^, (**c**) 200 mA/cm^2^, and (**d**) 300 mA/cm^2^.

As shown in Figure 3, the tensile fracture morphologies under different hydrogen-charging current densities exhibit pronounced necking and ductile fracture characteristics, with dimple-like microstructures observed on the fracture surfaces, indicating typical ductile fracture behavior. As the hydrogen-charging current density increases, the depth of the dimples gradually decreases, while their number and the proportion of smaller dimples progressively increase. In particular, the sample subjected to a hydrogen-charging current density of 300 mA/cm^2^ shows the smallest dimples with the highest density. Further, in conjunction with Figure 3d, each image was analyzed to quantify the number and size of dimples. When the hydrogen-charging current density increased from 0 to 300 mA/cm^2^, the number of dimples increased from 292 to 405. Meanwhile, the proportion of dimples with diameters in the range of 2–3 μm increased from 51.37% to 61.73%, and the average dimple diameter decreased from 5.21 μm to 4.47 μm. These statistical results further corroborate the aforementioned analysis. Overall, the dimples on the fracture surfaces become increasingly smaller and denser, suggesting a further reduction in the maximal elongation of the samples. The experimental results of other researchers have also demonstrated that with the gradual increase in hydrogen content, the fracture morphology transitions towards brittle fracture, indicating that the hydrides contributing to the fracture of zirconium alloys are of a brittle nature [2,3,15,34].

### 3.3. Characterization of Zirconium Hydride

**Figure 4:**
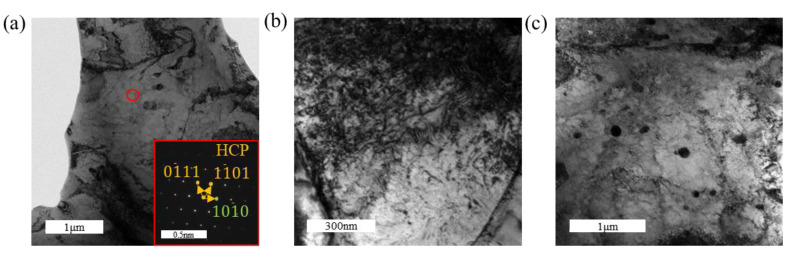
TEM images of post-tensile specimens under a hydrogen-charging current density of 0 mA/cm^2^: (**a**) the matrix; (**b**) dislocation entanglement; (**c**) second phases.

**Figure 5:**
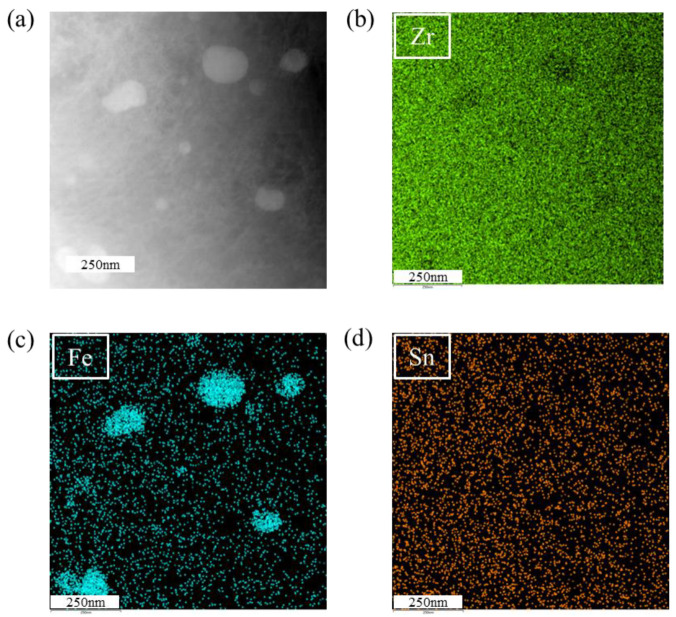
EDS analysis of the second phase. (**a**) bright-field image; (**b**–**d**) elemental mapping.

To further identify the microstructural characteristics of the corresponding samples, TEM was employed to observe the microstructures of tensile-fractured samples under different hydrogen-charging current densities. The microstructure of the sample under a hydrogen-charging current density of 0 mA/cm^2^ is shown in Figure 4, which indicates that no hydrides are formed. The matrix consists of α-Zr, with the presence of a second phase observed. Energy-dispersive spectroscopy (EDS) analysis, as shown in Figure 5, confirms that the second phase corresponds to iron precipitates, while other elements are uniformly distributed within the matrix. The second phase can impede dislocation motion, thereby enhancing the tensile strength of the zirconium alloy. However, no definitive evidence suggests a direct relationship between the second phase and the fracture behavior. The fracture mechanism is attributed to dislocation slip during tensile deformation, with dislocations accumulating near grain boundaries. This accumulation leads to the formation of an initial crack, ultimately resulting in fracture. In addition, the interaction between dislocation slip and twinning jointly facilitates crack formation, ultimately leading to fracture [35,36].

**Figure 6:**
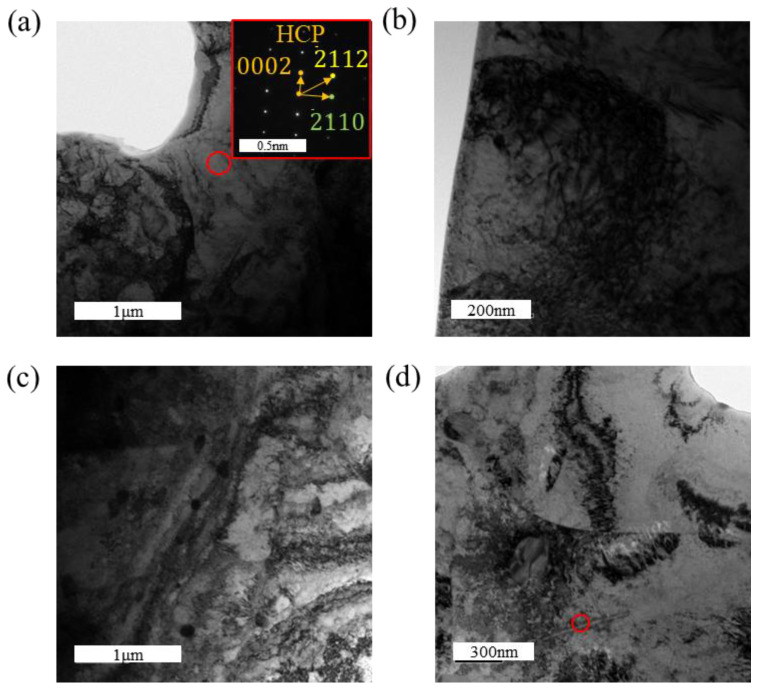
TEM images of post-tensile specimens under a hydrogen-charging current density of 100 mA/cm^2^: (**a**) the matrix; (**b**) dislocation entanglement; (**c**) second phases; (**d**) needle-like hydrides.

**Figure 7:**
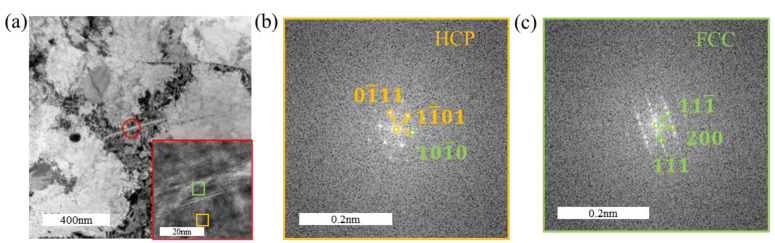
Morphology and structural analysis of hydrides in the tensile specimen under a hydrogen-charging current density of 100 mA/cm^2^: (**a**) matrix morphology with hydrides; (**b**) FFT pattern of the α-Zr matrix ; (**c**) FFT pattern of the zirconium hydride. The orientation relationship between the zirconium matrix and the hydride in the HRTEM image: ${\left(10\bar{1}0\right)}_{\alpha }//{\left(200\right)}_{\delta }$ and ${\left[1\bar{2}1\bar{3}\right]}_{\alpha }//{\left[011\right]}_{\delta }$.

Figure 6 presents the TEM microstructural features of the zirconium alloy following tensile fracture under a hydrogen-charging current density of 100 mA/cm^2^, providing insights into its microscopic characteristics. Figure 6c shows the formation of hydrides within the material, although the number of hydrides is extremely limited within the observed field of view. These hydrides exhibit a slender, needle-like morphology. High-resolution transmission electron microscopy (HRTEM) images reveal a certain orientation relationship between the hydrides and the α-Zr matrix. The fast Fourier transform (FFT) patterns of the hydrides and α-Zr matrix are shown in Figure 7b,c, respectively. Based on the FFT analysis, the hydrides are identified as δ-phase hydrides with a face-centered cubic (FCC) structure. The formation of δ-phase hydrides is accompanied by a 17% volumetric expansion, which can induce significant deformation fields near the hydride–matrix interface. Additionally, the interactions between δ-phase hydrides can promote the formation of localized plastic deformation, thereby accelerating the fracture of hydrides [2,37,38,39].

**Figure 8:**
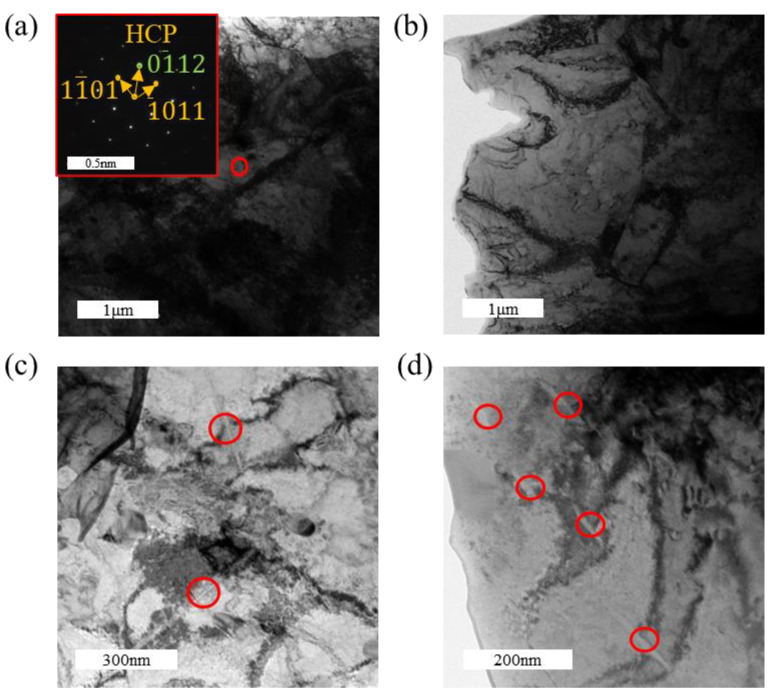
TEM images of post-tensile specimen under a hydrogen-charging current density of 200 mA/cm^2^: (**a**) the matrix; (**b**) second phases; (**c**,**d**) hydrides with distinct morphologies.

**Figure 9:**
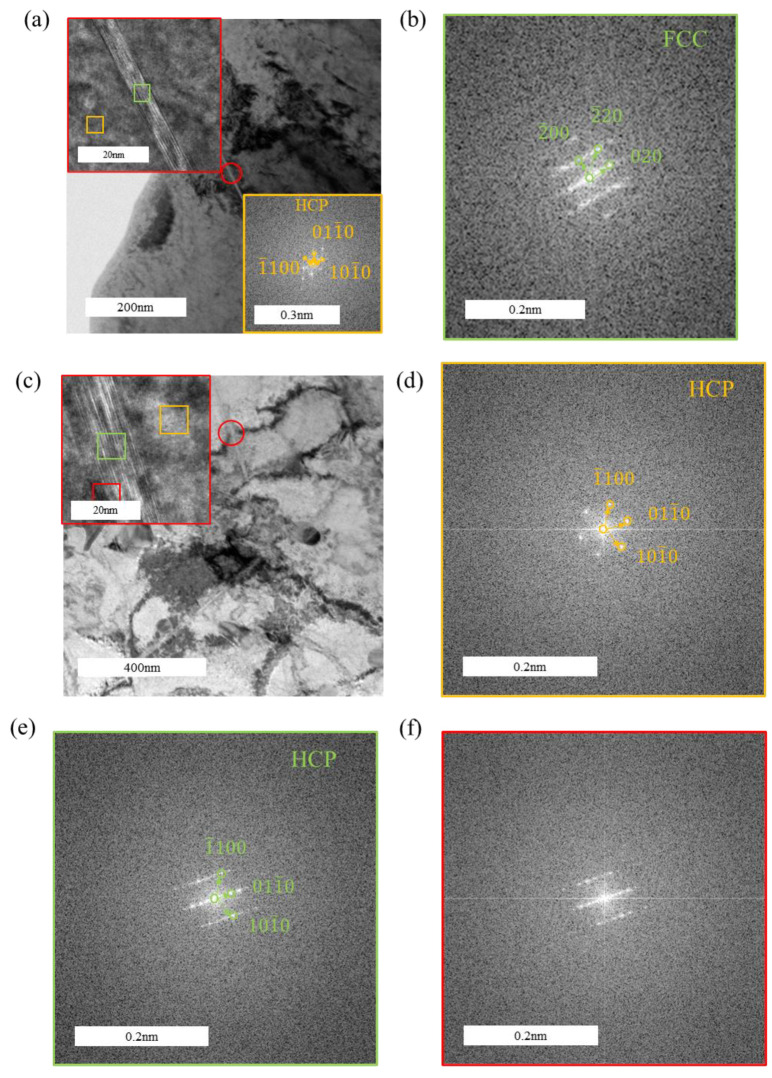
Morphology and structural analysis of slender needle-like hydrides in the tensile specimen under a hydrogen-charging current density of 200 mA/cm^2^: (**a**,**c**) matrix morphology with hydrides; (**b**,**e**) FFT pattern of the hydride; (**d**) FFT pattern of the α-Zr matrix; (**f**) overlaid FFT pattern of zirconium hydrides and the α-Zr matrix.

**Figure 10:**
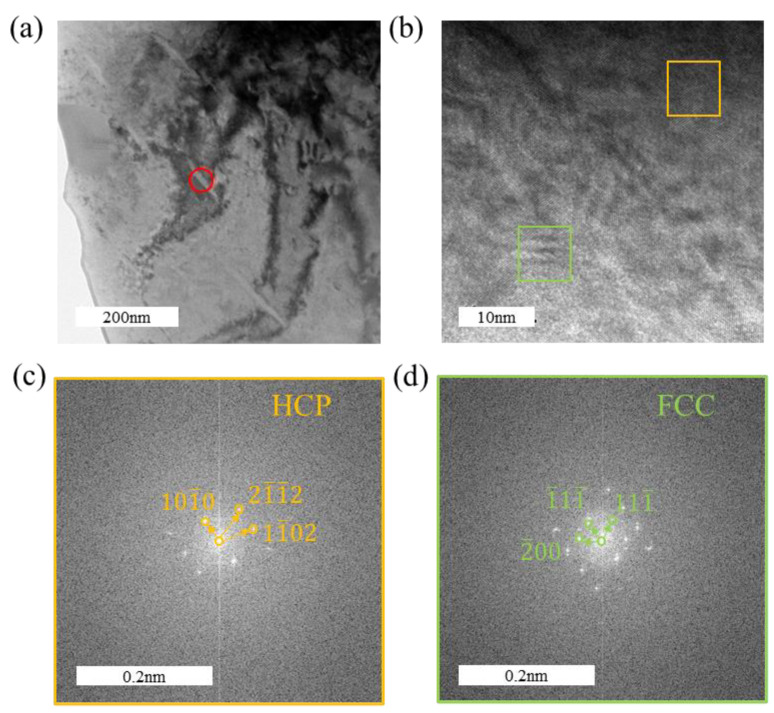
Morphology and structural analysis of short rod-like hydrides in the tensile specimen under a hydrogen-charging current density of 200 mA/cm^2^: (**a**) matrix morphology with hydrides; (**b**) HRTEM image of a hydride; (**c**) FFT pattern of the α-Zr matrix; (**d**) FFT pattern of the hydride.

Figure 8 presents the TEM images of the tensile-fractured sample under a hydrogen-charging current density of 200 mA/cm^2^. Figure 8c,d reveal the formation of hydrides with distinctly different lengths, and their quantity is noticeably higher compared to the sample charged at 100 mA/cm^2^. The hydrides shown in Figure 6d appear as slender needle-like structures, whereas the hydrides in Figure 8d are significantly shorter, indicating the formation of another type of fine rod-like hydrides. HRTEM characterization was performed on these two types of hydrides, as shown in Figure 9 and Figure 10, respectively. Figure 9 demonstrates that all observed hydrides are slender needle-like structures. However, fast Fourier transform (FFT) analysis of Figure 9a,c reveals that the needle-like hydrides consist of two types: one with a face-centered cubic (FCC) structure, identified as δ-phase hydrides, and the other with a hexagonal close-packed (HCP) structure. According to the FFT pattern of the hydride–matrix interface in Figure 9f, the HCP hydrides exhibit a “hexagonal–hexagonal” orientation relationship with the α-Zr matrix, characterized by parallel alignment between the basal plane of the hydride and the prismatic plane of the matrix, having the same crystallographic indices. For the hydride shown in Figure 9e, its lattice constants a and b are similar to those of the α-Zr matrix, while the c-axis length is approximately twice that of the α-Zr matrix, identifying it as ζ-phase hydride [30,40].

For the short rod-like hydrides shown in Figure 10, FFT analysis confirms that they possess an FCC structure, consistent with δ-phase hydrides. Due to the increased number of hydrides and the presence of ζ-phase hydrides, which are considered ductile hydrides, the maximal elongation of the sample during the tensile test decreases only slightly when the hydrogen-charging current density increases from 100 mA/cm^2^ to 200 mA/cm^2^.

In Figure 9a, the orientation relationship between the zirconium matrix and the hydride in the HRTEM image is ${\left(10\bar{1}0\right)}_{\alpha }//{\left(200\right)}_{\delta }$ and ${\left[1\bar{2}1\bar{3}\right]}_{\alpha }//{\left[011\right]}_{\delta }$. In Figure 9c, the orientation relationship between the zirconium matrix and the hydride is ${\left(10\bar{1}0\right)}_{\alpha }//{\left(10\bar{1}0\right)}_{\delta }$ and ${\left[1\bar{2}1\bar{3}\right]}_{\alpha }//{\left[1\bar{2}1\bar{3}\right]}_{\delta }$. In Figure 10, the orientation relationship between the zirconium matrix and the hydride in the HRTEM image is ${\left(10\bar{1}0\right)}_{\alpha }//{\left(200\right)}_{\delta }$ and ${\left[\bar{2}4\bar{2}3\right]}_{\alpha }//{\left[011\right]}_{\delta }$.

**Figure 11:**
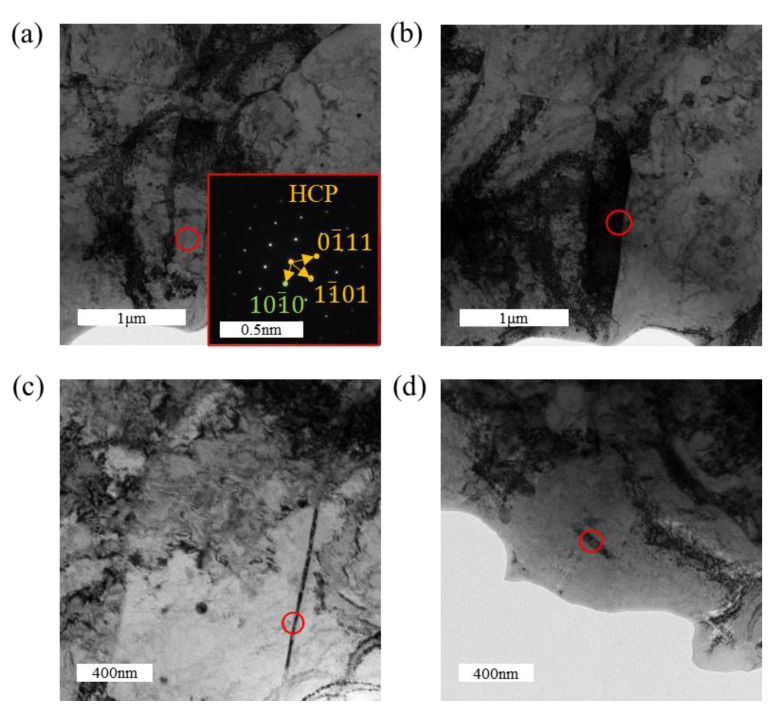
TEM images of the tensile specimen under a hydrogen-charging current density of 300 mA/cm^2^: (**a**) bright-field image of the matrix; (**b**–**d**) hydrides with various morphologies.

**Figure 12:**
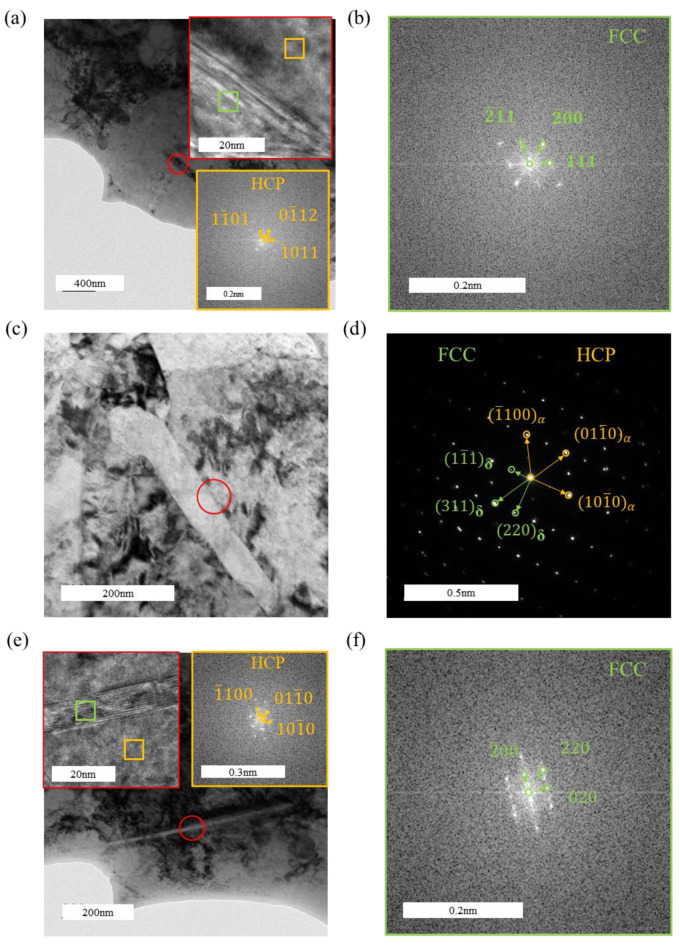
Morphology and structural analysis of lath-like and needle-like hydrides in the tensile specimen under a hydrogen-charging current density of 300 mA/cm^2^: (**a**,**c**,**e**) matrix morphology with hydrides; (**b**,**f**) FFT pattern of the hydride; (**d**) diffraction pattern of the hydride and zirconium matrix.

Figure 11 presents the TEM images of the tensile-fractured sample under a hydrogen-charging current density of 300 mA/cm^2^. Combined with Figure 11b–d, three distinct types of hydrides are identified: lath-like, slender needle-like, and short rod-like hydrides. Among these, the hydrides are predominantly lath-like and slender needle-like, while the short rod-like hydrides are scarce. HRTEM characterization of these three types of hydrides is shown in Figure 12. In Figure 12b, FFT analysis of the short rod-like hydride indicates an FCC structure, identified as δ-phase hydride. Similarly, Figure 12c–f show that both the lath-like and slender needle-like hydrides also possess an FCC structure, corresponding to the δ-phase hydride. It can be inferred that the formation sequence of hydrides changes with an increasing hydrogen-charging current density: short rod-like hydrides form first, which then transform into slender needle-like hydrides as the hydrogen-charging current density increases further, and ultimately evolve into lath-like hydrides at higher current densities. With increasing hydrogen-charging current density, the metastable ζ-phase hydrides, being thermodynamically unstable, also transform into δ-phase hydrides, further increasing the overall hydride content [41]. The formation of hydrides induces volumetric expansion, which generates compressive stress from the surrounding grains on the hydrides. The lath-like hydrides produce greater compressive stress compared to other morphologies, thereby further enhancing the tensile strength of the material [28]. It has been reported that at higher hydrogen concentrations, lath-like hydrides become more prominent and may form twinned structures, resulting in lattice mismatch and an increased stress concentration [42]. The increased amount of brittle hydrides ultimately leads to the earlier failure of the specimens.

In Figure 12a, the orientation relationship between the zirconium matrix and the hydride in the HRTEM image is ${\left(1\bar{1}01\right)}_{\alpha }//{\left(\bar{2}\bar{1}1\right)}_{\delta }$ and ${\left[01\bar{1}1\right]}_{\alpha }//{\left[011\right]}_{\delta }$. In Figure 12d, the orientation relationship between the zirconium matrix and the hydride in the HRTEM image is ${\left(10\bar{1}0\right)}_{\alpha }//{\left(220\right)}_{\delta }$ and ${\left[0001\right]}_{\alpha }//{\left[011\right]}_{\delta }$. In Figure 12e, the orientation relationship between the zirconium matrix and the hydride in the HRTEM image is ${\left(10\bar{1}0\right)}_{\alpha }//{\left(\bar{2}00\right)}_{\delta }$ and ${\left[0001\right]}_{\alpha }//{\left[001\right]}_{\delta }$.

**Figure 13:**
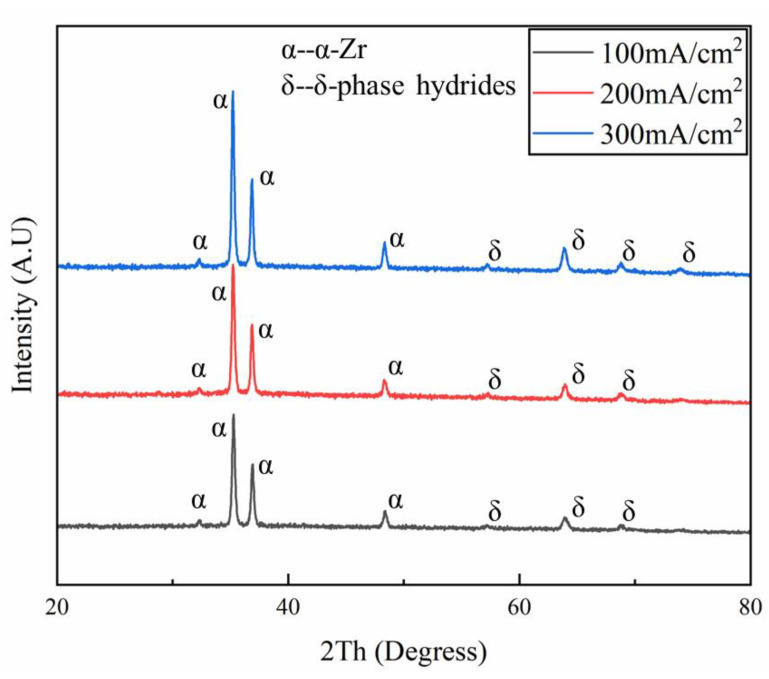
XRD spectra of specimen after tensile fracture under different hydrogen-charging current densities. The black, red, and blue lines represent the experimental results under hydrogen-charging current densities of 100, 200, and 300 mA/cm^2^, respectively. α denotes the zirconium matrix, while δ represents the δ-hydride phase.

To further determine the changes in the type and quantity of hydrides caused by the increase in hydrogen-charging current density, an X-ray diffractometer (XRD) was used to analyze the phase structures of tensile-fractured samples under different hydrogen-charging current densities. The XRD results of the tensile fracture samples with varying characteristics are shown in Figure 13. From Figure 13, it can be observed that δ-phase hydrides formed in the samples when the hydrogen-charging current densities were 100, 200, and 300 mA/cm^2^. As the hydrogen-charging current density increased, the peak intensity corresponding to the δ-phase hydrides increased, and the diffraction peak area also gradually expanded, indicating that the quantity of hydrides with this crystal orientation increased in the zirconium alloy. On the other hand, ζ-phase hydrides maintain a completely coherent relationship with the zirconium matrix, making their diffraction signals easily overshadowed by the matrix characteristics in the XRD results. Therefore, ζ-phase hydrides are not displayed in the XRD spectra [30,43].

The analysis of hydride types in zirconium alloys after tensile fracture under different hydrogen-charging current densities reveals that, at low temperatures, two primary types of hydrides are present: ζ-phase hydrides and δ-phase hydrides. The ζ-phase hydrides predominantly appear as slender needle-like structures. However, ζ-phase hydrides are metastable, and their influence diminishes with increasing hydrogen-charging current density, potentially transforming into δ-phase hydrides. In contrast, δ-phase hydrides are consistently present and exhibit diverse morphologies under different hydrogen-charging current densities. These morphologies include short rod-like, slender needle-like, and lath-like structures, with varying degrees of dislocation accumulation observed in their vicinity. As the hydrogen-charging current density increases, the quantity of δ-phase hydrides further increases, forming twinned lath-like hydrides that exacerbate stress concentration. Consequently, at a hydrogen-charging current density of 300 mA/cm^2^, the maximal elongation of the zirconium alloy exhibits a precipitous decline.

## 4. Conclusions

In this study, a novel zirconium alloy tube for nuclear fuel cladding was designed, and its fracture behavior in different hydrogen environments was investigated.

The influence of hydrides on the fracture behavior of the zirconium alloy was studied under hydrogen-charging current densities ranging from 0 to 300 mA/cm^2^. At room temperature, all samples exhibited ductile fracture. However, its tensile strength increases with the rise in hydrogen-charging current density, while the maximal elongation exhibits a decreasing trend. Specifically, when the hydrogen-charging current density increases from 200 mA/cm^2^ to 300 mA/cm^2^, the tensile strength rises from 530.96 MPa to 550.76 MPa, whereas the maximal elongation decreases from 20.77% to 15.18%, indicating a significant reduction in hydrogen embrittlement resistance. This is attributed to the change in hydride types. Currently, the standard zirconium alloy commonly reported in the literature is Zry-4, which exhibits a tensile strength of approximately 450–500 MPa and an elongation of 20–30% at room temperature. However, as the hydrogen content gradually increases, a significant ductile-to-brittle transition occurs. In contrast, the novel zirconium alloy studied here demonstrates an elongation comparable to Zry-4 at room temperature, while achieving an approximately 5% improvement in tensile strength. Furthermore, under hydrogen environments during tensile testing, the novel alloy exhibits a smaller reduction in elongation and does not undergo brittle fracture [34,37,44,45,46].

Under different hydrogen-charging current densities, the morphology and structure of the hydrides varied, manifesting as short rod-like, slender needle-like, and lath-like structures. Two types of hydrides were identified: ζ-phase hydrides and δ-phase hydrides, which ultimately influenced the mechanical properties of the samples. At a hydrogen-charging current density of 300 mA/cm^2^, all hydrides are of the FCC-structured δ-phase, predominantly exhibiting lath-like and slender needle-like morphologies. Among these, the lath-like hydrides generate higher compressive stress, contributing to the enhancement of the tensile strength of the zirconium alloy. The lath-like hydrides adopt a twinned structure, which intensifies stress concentration, leading to earlier fracture. This indicates that with the increase in hydrogen-charging current density, the morphologies of the hydrides gradually evolve into slender needle-like and lath-like structures, while the hydride type completely transitions to the δ-phase. This further enhances the tensile strength of the zirconium alloy while significantly reducing its elongation.

## Data Availability

The original contributions presented in this study are included in the article. Further inquiries can be directed to the corresponding author.

## References

[1] Coleman C.E., Hardie D. (1966). The hydrogen embrittlement of α-zirconium—A review. J. Less Common Met..

[2] Marashi S., Abdolvand H. (2024). The micromechanics of fracture of zirconium hydrides. Acta Mater..

[3] Kolesnik M. (2024). Micro-mechanisms of the ductile-to-brittle transition in hydrogenated zirconium alloys: A review and a comparison analysis of experimental data and theoretical approaches. Eng. Fail. Anal..

[4] Hong X., Ma F.Q., Zhang J., Du D., Tian H., Xu Q., Zhou J., Gong W.J. (2024). Effect of hydride orientation on tensile properties and crack formation in zirconium alloy cladding tubes. J. Nucl. Mater..

[5] Zinkle S.J., Was G.S. (2013). Materials challenges in nuclear energy. Acta Mater..

[6] Duan Z., Yang H., Satoh Y., Murakami K., Kano S., Zhao Z., Shen J., Abe H. (2017). Current status of materials development of nuclear fuel cladding tubes for light water reactors. Nucl. Eng. Des..

[7] Lin X.-H., Beyerlein I.J., Han W.-Z. (2024). Annealing cracking in Zr and a Zr-alloy with low hydrogen concentration. J. Mater. Sci. Technol..

[8] Zhu X., Lin D.Y., Fang J., Gao X.-Y., Zhao Y.-F., Song H.-F. (2018). Structure and thermodynamic properties of zirconium hydrides by structure search method and first principles calculations. J. Comput. Mater. Sci..

[9] Bang S., Kim H.-A., Noh J.-S., Kim D., Keum K., Lee Y. (2022). Temperature-dependent axial mechanical properties of Zircaloy-4 with various hydrogen amounts and hydride orientations. Nucl. Eng. Technol..

[10] Narang P.P., Paul G.L., Taylor K.N.R. (1977). Location of hydrogen in a-zirconium. J. Less-Common Met..

[11] Wang Z., Garbe U., Li H., Wang Y., Studer A.J., Sun G., Harrison R.P., Liao X., Alvarez M.V., Santisteban J.R. (2014). Microstructure and texture analysis of δ-hydride precipitation in Zircaloy-4 materials by electron microscopy and neutron diffraction. J. Appl. Crystallogr..

[12] Wang H., Cheng X., Zhang Y.G., Wang M.M., Zhao B.L., Xie Z.M., Zhang T., Liu R., Wu X.B., Wang X.P. (2021). Hydrogen embrittlement of bulk W-0.5 wt% ZrC alloy induced by annealing in hydrogen atmosphere. J. Nucl. Mater..

[13] Bertolino G., Meyer G., Ipiña J.P. (2002). Degradation of the mechanical properties of Zircaloy-4 due to hydrogen embrittlement. J. Alloys Compd..

[14] Kim J.H., Lee M.H., Choi B.K., Jeong Y.H. (2007). Effect of the hydrogen contents on the circumferential mechanical properties of zirconium alloy claddings. J. Alloys Compd..

[15] Tung H.-M., Chen T.C., Tseng C.C. (2016). Effects of hydrogen contents on the mechanical properties of Zircaloy-4 sheets. Mater. Sci. Eng. A.

[16] Nagase F., Sugiyama T., Fuketa T. (2009). Optimized ring tensile test method andhydrogen effect on mechanical properties of Zircaloy cladding in hoopdirection. J. Nucl. Sci. Technol..

[17] Jia Y.-J., Han W.-Z. (2023). Mechanisms of Hydride Nucleation, Growth, Reorientation, and Embrittlement in Zirconium: A Review. Materials.

[18] Long F., Luo Y., Badr N.N., Shiman O., Topping M., Persaud S.Y., Yao Z., Béland L.K., Daymond M.R. (2021). Identifying the true structure and origin of the water-quench induced hydride phase in Zr-2.5Nb alloy. Acta Mater..

[19] Hsu H.-H., Tsay L.-W. (2011). Effect of hydride orientation on fracture toughness of Zircaloy-4 cladding. J. Nucl. Mater..

[20] Qin W., Szpunar J.A., Kozinski J. (2012). Hydride-induced degradation of hoop ductility in textured zirconium-alloy tubes: A theoretical analysis. Acta Mater..

[21] Qin W., Kumar N.A.P.K., Szpunar J.A., Kozinski J. (2011). Intergranular δ-hydride nucleation and orientation in zirconium alloys. Acta Mater..

[22] Li J., Li M., Guan B., Xin Y., Wu Y., Liu X., Chen G. (2022). Uncovering the hydride orientation-mediated hoop fatigue mechanism in a zirconium alloy cladding tube. Int. J. Plast..

[23] Hong E., Dunand D.C., Choe H. (2010). Hydrogen-induced transformation superplasticity in zirconium. Int. J. Hydrogen Energy.

[24] Steuwer A., Santisteban J.R., Preuss M., Peel M.J., Buslaps T., Harada M. (2009). Evidence of stress-induced hydrogen ordering in zirconium hydrides. Acta Mater..

[25] Liu S.-M., Han W.-Z. (2024). Effect of grain boundary character on intergranular hydrides precipitation in zirconium. Acta Mater..

[26] Thuinet L., Besson R. (2012). Ab initio study of competitive hydride formation in zirconium alloys. Intermetallics.

[27] Chernikov A.S., Syasin V.A., Kostin V.M., Boiko E.B. (2002). Influence of hydrogen content on the strength and the presence of defects in ε-zirconium hydride. J. Alloys Compd..

[28] Wang S., Li S., Li R., Wang Y., Xu N., Xue F., Bai G., Wang Y.-D. (2020). Microscopic stress and crystallographic orientation of hydrides precipitated in Zr-1Nb-0.01Cu cladding tube investigated by high-energy X-ray diffraction and EBSD. J. Nucl. Mater..

[29] Huang X., Zhang B., Zhang X. (2024). Reconstructing brittle hydrides using pulsed electric current to restore the degraded performance of zirconium alloys. Int. J. Hydrogen Energy.

[30] Li L.S., Wang K.Y., Che F.Z., Liu G., Ren Y., Wang Y. (2017). Investigations of deformation-induced δ→ζ phase transformation in zirconium hydride by in situ high-energy X-ray diffraction. Acta Mater..

[31] Shi H., Li J., Mao J., Lu W. (2019). The Elimination of the Yield Point Phenomenon in a New Zirconium Alloy: Influence of Degree of Recrystallization on the Tensile Properties. Scr. Mater..

[32] Muta H., Nishikane R., Ando Y., Matsunaga J., Sakamoto K., Harjo S., Kawasaki T., Ohishi Y., Kurosaki K., Yamanaka S. (2018). Effect of hydrogenation conditions on the microstructure and mechanical properties of zirconium hydride. J. Nucl. Mater..

[33] Zhao C., Song X., Yang Y., Zhang B. (2013). Hydrogen absorption cracking of zirconium alloy in the application of nuclear industry. Int. J. Hydrogen Energy.

[34] Sun H., Zhou H., Luan B., Zhang Y., Zhu X., Xu C., Sun C., Murty K.L., Fan G., Liu Q. (2024). In-situ EBSD analysis of hydride phase transformation and its effect on micromechanical behavior in Zircaloy-4 under uniaxial tensile loading. J. Mater. Res. Technol..

[35] Capolungo L., Beyerlein I.J., Kaschner G.C., Tomé C.N. (2009). On the interaction between slip dislocations and twins in HCP Zr. Mater. Sci. Eng. A.

[36] Li J., Liu A., Liu X., Ye X., Wang J., Zhang Y., Zhang Z. (2025). An investigation of slip and twinning behavior of a zirconium alloy during plastic deformation based on in-situ SEM-EBSD. J. Alloys Compd..

[37] Birch R.M., Douglas J.O., Britton T.B. (2023). Characterization of local deformation around hydrides in Zircaloy-4 using conventional and high angular resolution electron backscatter diffraction. Mater. Charact..

[38] Qin W., Szpunar J.A., Kozinski J. (2015). Hydride-induced degradationof zirconium alloys: A criterion for complete ductile-to-brittle transition and itsdependence on microstructure. Proc. R. Soc. A.

[39] Wang S., Giuliani F., Britton T.B. (2019). Microstructure and formation mechanisms of δ-hydrides in variable grain size Zircaloy-4 studied by electron backscatter diffraction. Acta Mater..

[40] Yang Z., Li F., Yao J., Li S., Wang Y. (2023). The HRTEM characterization of electropolishing-induced ζ-hydride in the α-Zr/δ-hydride interface in Zircaloy-4. Mater. Lett..

[41] Wang S., Li S., Zhang X., Liang S., Wang Y., Gong W., Ren Y., Wang Y.-D. (2024). Unveiling the formation of nanotwin-mediated metastable ζ-hydrides in fatigued Zr-1Nb-0.01Cu cladding tube. Acta Mater..

[42] Tan X., Liu Y., Li F., Qiu R., Liu Q. (2023). Formation of nanocrystalline γ-ZrH in Zr-Nb alloy: Crystal structure and twinning. Micron.

[43] Maimaitiyili T., Bjerken C., Steuwer A., Wang Z., Daniels J., Andrieux J., Blomqvist J., Zanellato O. (2017). In situ observation of γ-ZrH formation by X-ray diffraction. J. Alloys Compd..

[44] Torres J.R., Mizzi C.A., Rehn D.A., Smith T., Paisner S.W., Terricabras A.J., Parkison D.M., Vogel S.C., Kohnert C.A., Hayne M.L. (2025). High-temperature structure; elasticity, and thermal expansion of ε-ZrH1.8. J. Nucl. Mater..

[45] Zhao Z., Morniroli J.P., Legris A., Ambard A., Khin Y., Legras L., Blat-Yrieix M. (2008). Identification and characterization of anew zirconium hydride. J. Microsc..

[46] Deng S., Song H., Liu H., Zhang S.-H. (2021). Effect of uniaxial loading direction on mechanical responses and texture evolution in cold pilgered Zircaloy-4 tube: Experiments and modeling. Int. J. Solids Struct..

